# Obesity, DNA Damage, and Development of Obesity-Related Diseases

**DOI:** 10.3390/ijms20051146

**Published:** 2019-03-06

**Authors:** Marta Włodarczyk, Grażyna Nowicka

**Affiliations:** 1 Department of Biochemistry and Pharmacogenomics, Faculty of Pharmacy with Division of Laboratory Medicine, Medical University of Warsaw, Banacha 1B, 02-097 Warsaw, Poland; grazyna.nowicka@wum.edu.pl; 2 Centre for Preclinical Research, Medical University of Warsaw, Banacha 1B, 02-097 Warsaw, Poland

**Keywords:** DNA damage, obesity, inflammation, oxidative stress, ROS, cancer

## Abstract

Obesity has been recognized to increase the risk of such diseases as cardiovascular diseases, diabetes, and cancer. It indicates that obesity can impact genome stability. Oxidative stress and inflammation, commonly occurring in obesity, can induce DNA damage and inhibit DNA repair mechanisms. Accumulation of DNA damage can lead to an enhanced mutation rate and can alter gene expression resulting in disturbances in cell metabolism. Obesity-associated DNA damage can promote cancer growth by favoring cancer cell proliferation and migration, and resistance to apoptosis. Estimation of the DNA damage and/or disturbances in DNA repair could be potentially useful in the risk assessment and prevention of obesity-associated metabolic disorders as well as cancers. DNA damage in people with obesity appears to be reversible and both weight loss and improvement of dietary habits and diet composition can affect genome stability.

## 1. Introduction

The rising prevalence of obesity has become a major health problem in adults, as well as among children and adolescents. Obesity is a complex chronic disease, characterized by a significant increase in body fat tissue mass, and it is associated with disturbances in lipid and glucose metabolism, chronic inflammation and oxidative stress, and an increased risk of several diseases, most notably cardiovascular diseases, diabetes, and cancers, and with a decrease in life expectancy [1,2,3]. In people with obesity accumulation of DNA damage has been reported and suggested to be involved in the development of obesity-related disease [4,5,6]. DNA lesions have an impact on DNA replication, leading to mutations and thus may create a hazard for cell metabolism and cell survival [7]. Body weight loss has been found to result in a reduction in the level of DNA damage [8].

The aim of this paper is to underline obesity as DNA damaging factor and to present the relationship between obesity, DNA damage and development of metabolic disorders, and cancer.

## 2. Inflammation and Reactive Oxygen Species (ROS)-Induced DNA Damage

Inflammation is activated to protect the body against these harmful stimuli [9]. Chronic inflammation has been linked to aging and numerous chronic diseases such as cardiovascular diseases, autoimmune diseases, and cancer [10,11]. Proinflammatory signal recruits and activates neutrophils and macrophages and in turn, endogenous oxygen and nitrogen species are created. Moreover, reactive oxygen species (ROS) are also formed in cells during mitochondrial oxidative metabolism, apoptosis or the enzymatic reaction of nicotinamide adenine dinucleotide phosphate (NADPH) oxidases, superoxide dismutase (SOD), myeloperoxidase (MPO) and nitric oxide synthase (NOS) [12].

Despite the presence of the specific defense system against radicals, constant ROS production and low antioxidant activity can lead to the loss of balance between the formation of ROS and the operation of a protective system, resulted in the development of oxidative stress. The increased ROS production and oxidative stress may induce endogenous DNA damage, transcription interruption and induce cell-cycle arrest [13,14]. Lipid peroxidation processes are also induced by ROS and lead to the formation of DNA reactive lesions [15].

Among ROS, free radicals such as superoxide or hydroxyl radical are the most hazardous. The superoxide (O_2_ •−) is generated during aerobic respiration and hydrogen peroxide (H_2_O_2_) during dismutation of superoxide by superoxide dismutase. In addition, several oxidases can also produce hydrogen peroxide [16]. Hydroxyl radical (HO•) can be formed as a result of both Fenton reaction and Weber-Weiss reaction [17]. Activated macrophages and neutrophils involved in inflammation generate oxidants as peroxynitrite (ONOO−) and nitrosoperoxycarbonate (ONOOCO_2_−), hypohalous acids (HOCl, HOBr), and nitrosating agent (N_2_O_3_) [18]. Furthermore, ROS can participate in lipid peroxidation and generated products such as etheno-, propano-, and malondialdehyde interact with DNA, form DNA adducts and damage DNA structure [14,19]. ROS attack can lead to base lesions such as oxidation, alkylation, methylation, nitration, deamination and single or double-strand DNA breaks, or cross-links in DNA structure. The frequently occurred DNA lesions caused by ROS are 8-hydroxyguanine, 7,8-dihydro-8-oxoguanine (commonly termed 8-oxoguanine: 8-OHdG), thymine glycol, Fapy Ade (4,6-diamino-5-formamidopyrimidine) and Fapy Gua (2,6-diamino-4-hydroxy-5-formamidopyrimidine) [20]. The occurrence of DNA lesions can induce mutations during DNA replication, as 8-OHdG can cause a change in GC to TA (transversion), resulting in mutagenesis and cancer initiation [20,21].

## 3. DNA Damage Repair

The DNA repair system exists to overcome DNA damage and maintain the integrity of the DNA structure. In general, DNA damage repair process involves the recognition of DNA damage by specific sensors, generation, and amplification of the DNA damage signal, transduction of this signal to the cytoplasm and activation of specific effectors [21]. Inter-individual variations in the activity of enzymes that participate in DNA repair pathways have been described [22,23]. Therefore, some differences in the efficiency of DNA repair and observed levels of endogenous DNA damage can be expected [24,25].

**Figure 1:**
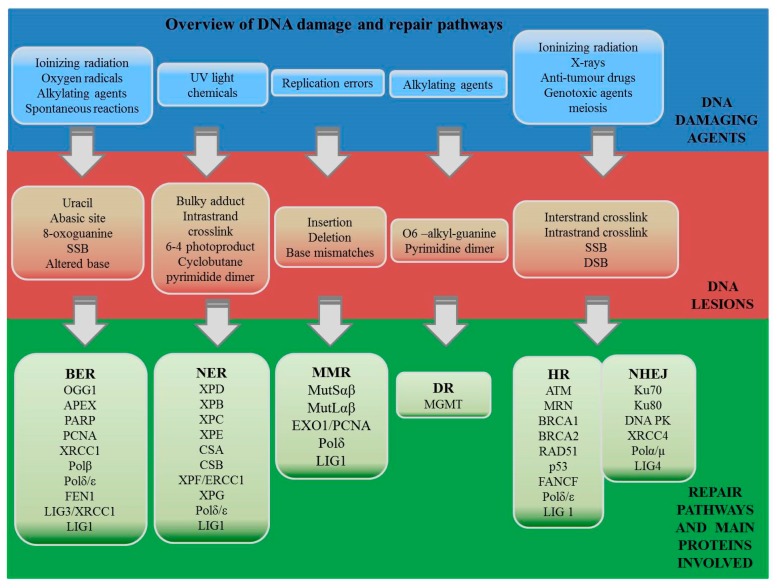
Overview of DNA damaging agents, induced DNA lesions, and their repair pathways (BER—base excision repair, NER—nucleotide excision repair, MMR—mismatch repair, DR—direct repair, NHEJ—non-homologous end-joining; and HR—homologous recombination). Shortcuts are explained in the abbreviation section.

Among several known DNA repair mechanisms, direct repair occurs during the replication, while indirect repair takes place after the DNA synthesis [26]. The indirect repair strategy includes base excision repair (BER), nucleotide excision repair (NER), mismatch repair (MMR), the non-homologous end-joining (NHEJ) and homologous recombination (HR) pathways. Explanation of the pathways involved in DNA repair by three scientists was recognized by award them the Nobel Prize in chemistry in 2015 [27]. Tomas Lindahl described the model of BER, which is involved in modified bases repair [28]. Paul Modrich discovered a distinct pathway that detects and removes bases that are misincorporated during DNA replication. Finally, Aziz Sancar proposed the mechanism for removal of DNA adducts NER [29]. Overview of DNA damaging agents, induced DNA lesions, and their repair pathways is presented in Figure 1.

## 4. Obesity and DNA Damage

In people with obesity, a broad range of DNA lesions such as double strand breaks (DSB), single strand breaks (SSB) or oxidized bases and about 2-times higher DNA damage in lymphocytes than in normal weight subjects have been observed and a correlation between body–mass index (BMI) and DNA damage was also found [5,30,31]. A significant difference in levels of DNA damage measured by H2AX phosphorylation was also observed in children with overweight and obesity compared to lean controls [32]. Lymphocytes from people with obesity had more mitomycin C-induced DNA damage compared to cells from normal weight subjects [33]. However, available data regarding the relationship between obesity and levels of oxidized bases in DNA such as 8-oxodG and 8-OHdG are inconsistent [34,35,36,37].

There is well accepted that in obesity chronic energy overload results in enhanced ROS production and inflammation [38]. Available data indicate that ROS source can differ depending on the stage of obesity [39]. In the early stages of obesity increased adipocyte uptake of glucose and fatty acids activates NOX4, the major NADPH oxidase isoform in adipocytes, and induces ROS production. NOX4 silencing was reported to decrease ROS generation and inhibition of monocyte chemoattractant protein-1 [40]. Excessive accumulation of fat in adipocytes promotes proinflammatory adipokines production. Proinflammatory cytokines induce invasion of the target tissue by immune cells and development of chronic inflammation [41]. Accumulation of T-lymphocytes and macrophages in adipose tissue during obesity development promote ROS production by NOX2, the NADPH oxidase expressed in inflammatory cells. In addition, adipocytes and smooth muscle cells exposed to high FFA (free fatty acid) or high glucose concentrations showed increased mitochondrial fission and increased mitochondrial ROS production [42,43,44]. Characteristic for obesity excessive accumulation of triglycerides in adipocytes results in enhanced mitochondrial β-oxidation of FFA and increased mitochondrial ROS generation.

Furthermore, chronic inflammation associated with obesity is strongly involved in the formation of DNA lesion [38]. Activated macrophages secrete cytokines such as TNFα, and IL-6 which can induce DNA damage in non-targeted tissue distant from the site of inflammation [45,46]. Released cytokines can travel to different regions of the body and activate resident macrophages to produce proinflammatory molecules such as COX2, NOS, superoxide, ROS, and NO [47,48]. The release of these molecules can lead to oxidative DNA damage in cells. Also, macrophages that must absorb apoptotic cells can move to another region of the body and then release factors inducing DNA damage [49]. The translocation of DNA damaging factors via macrophages can deliver a high amount of damage-inducing signals from distant sites and can also be specific to regions where the macrophages are likely to travel (e.g., gut, spleen, skin, lymph nodes). Thus, obesity-associated oxidative stress and inflammation can induce DNA damage in different tissues.

Published intervention weight loss trails in obese in which DNA damage assessment was performed are limited. A significant decrease in levels of DNA damage was found after a low caloric diet-induced weight loss [8,50]. Improvement of genomic stability, characterized by a reduction of oxidative damage in saliva, was observed also after bariatric surgery associated weight loss in patients with morbid obesity [51].

### 4.1. Obesity and DNA Damage Repair

**Figure 2:**
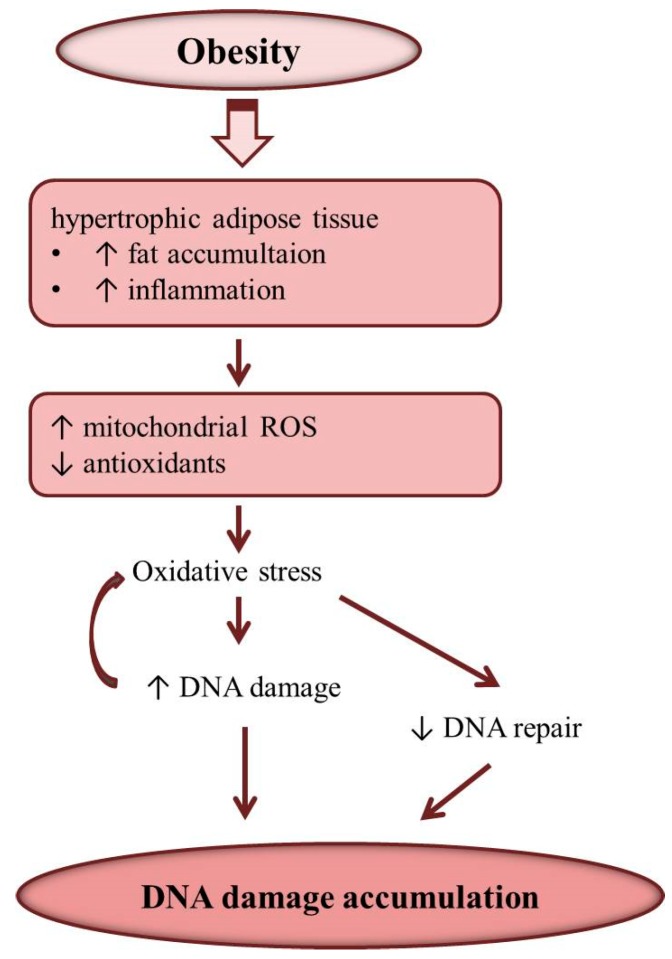
Obesity and DNA damage. Obesity is associated with inflammation and oxidative stress which induces DNA damage and inhibits DNA damage repair resulting in the accumulation of DNA damage in adipocyte and other tissues.

Disturbances in DNA damage response pathway related to enhanced increased body weight were reported [52]. An inverse association between BMI and nucleotide excision repair (NER) capacity was found in young females [53]. Presence of obesity was also recognized to alter the repair of DSBs induced by genotoxic agents [34]. Obesity-associated enhanced ROS production can modulate the DNA damage response through the impact on the expressions of genes involved in DNA repair (Figure 2) [54,55]. Inhibition of DNA repair enzymes provoked by the oxidative stress has been reported [56,57]. In obese alter expression of genes related to response to stress and toxic agents were also recognized [58].

It can be hypothesized that epigenetic mechanisms might be involved in the regulation of genes encoding DNA repair proteins. Unbalanced and high-fat diet commonly observed in overweight and obese subjects can alter methyl group availability and disturb epigenetic regulation of DNA repair genes. Enhanced dietary fat consumption was reported to significantly alter DNA methylation and gene expression [59,60]. Recently high-fat diet was also found to suppress DNA damage repair by increasing lysine homocysteination in proteins involved in DNA damage repair [61]. Low intakes of vitamins C and E, as well as vitamins B and zinc, were reported to be associated with enhanced DNA damage [62,63]. In obese women, daily intakes of vitamins C and E were recognized to significantly affect DNA damage in lymphocytes indicating that in obese adequate vitamins C and E consumption could reduce levels of basal DNA damage probably, by the antioxidant effect of this vitamins [5]. However, in obese mice, an active antioxidant, EGCG (epigallocatechin-3-gallate) was shown to enhance methylation of MGMT (O6-Methylguanine-DNA methyltransferase) and MLH1 (MutL homolog 1) genes involved in direct and mismatch DNA repair, respectively [64]. Reduction of *MLH1* gene expression was also observed and methylation rate of *MLH1* was related to DNA damage in mice on high-fat diet supporting the role of epigenetic mechanisms in the regulation of DNA damage response [65].

### 4.2. Obesity and Mitochondrial DNA Damage

DNA lesions occur in both nuclear and mitochondrial DNA (mtDNA). Because of the absence of nucleotide excision repair mechanism in mitochondria, mtDNA is more susceptible to damage caused by reactive species than nuclear DNA. Photodimers and bulky adducts arising as a result of oxidative stress related to inflammation and environmental factors are not efficiently removed from mtDNA [66,67]. Despite mtDNA encodes only 1% of the mitochondrial proteins, mitochondrial diseases are associated with a high number of lesions in mtDNA [68]. In addition, enhanced degradation of mtDNA and decreased mtDNA copy number are related also to diabetes, cancer or neurodegenerative diseases [69,70,71]. The accumulation of oxidative mtDNA lesions may result in rearrangements or point mutations which can be maternally inherited [72].

In obesity, mitochondrial dysfunction leads to failure in fatty acid (FA) oxidation and disturbances in glucose homeostasis [43,73,74]. Elevated urinary excretion of mtDNA observed in morbidly obese patients was found significantly reduced after bariatric surgery associated weight loss [75]. The animal study showed that in mice fed high-fat diet mtDNA damage increased and was associated with mitochondrial dysfunction [76]. Additionally, oxidized mtDNA was found to induce synthesis of proinflammatory cytokines such as IL-6, TNF-α, pro-IL-1β through the activation of toll-like receptor 9 (TLR-9) [77,78]. Therefore obesity-associated inflammation could be in part both a cause and a consequence of the accumulation of mtDNA lesions.

### 4.3. Effect of Parental Obesity-Related DNA Damage on Offspring

The obesity-associated DNA damage may at least in part be responsive for disturbances in reproductive capacity of obese subjects and their offspring’s health [79]. Adiposity was recognized to cause sperm DNA fragmentation, affect DNA methylation and cause aberrancies in chromatin in male germ cells [80,81]. In patients with obesity, high DNA fragmentation index (DFI) in sperm and reduced fertility was recognized [82,83]. DNA damage in germ cells may be a result of increased ROS production characteristic for obesity [59,84,85]. DNA damage was present in the daughter cells after subsequent cell division indicating ineffective DNA damage response [86]. Therefore, it is suspected that the appearance of DNA lesions in germ cells can be transmitted to the genome of future generations [87]. Maternal obesity may cause de novo mutations in the embryo, change the methylation status of the genes in embryo and through miRNA affects the expression of embryonic proteins [88,89,90,91].

## 5. DNA Damage and Obesity-Related Metabolic Disorders

Obesity is well recognized to be involved in the development of diabetes and atherosclerosis-related diseases and to increase the mortality rate, particularly deaths from cardiovascular diseases [92]. In mice and humans, mutations of genes related to the DNA repair result in phenotypic changes similar to those observed in obesity-associated metabolic and cardiovascular abnormalities [93]. The p53 protein, the transcriptional factor mediating the DNA damage response and involved in preserving genomic stability, has been shown to affect obesity-associated diseases [94]. DNA damage-induced p53 activation was found to be involved in aging-related diseases, since mice with a p53 mutant allele associated with p53 activation developed premature aging and mice overexpressing naturally occurring truncated p53 isoform have a short lifespan [95,96]. A higher mutation amount in the p53 gene was found in cancers [97]. In breast cancer enhanced BMI was related to p53 mutation [98]. Activation of p53 signaling in vessels, heart, and the visceral adipose tissue of obese was found to contribute to diabetes progression and atherosclerosis [99]. DNA damage in obese adipocytes can induce p53 pathway involved in altered metabolism of adipocytes. In consequences of adipocyte dysfunction, tissue inflammation and insulin resistance appear [100]. Under a high-calorie diet, the p53/p21-signaling pathway was found to be involved in adipocyte differentiation, hypertrophy, induction of inflammation, and development of systemic insulin resistance, which commonly occurs in obese patients. In mice overexpression of Δ40p53, p53 isoform alters the balance between the full-length and short isoforms and hyperactivates p53 resulting in increased p21 expression and developed of hypoinsulinemia, glucose intolerance, and diabetes [101]. Accumulation of DNA damage in pancreatic β-cell as well as in adipocytes results in cell senescence, which contributes to the development of disturbances in glucose metabolism and systemic insulin resistance [100,102]. Insulin resistance is an important underlying mechanism accelerating development of obesity-associated comorbidities [103]. The relationship between type 2 diabetes and DNA double-strand breaks was recognized. In people with obesity and diabetes, BMI was positively correlated with oxidative DNA damage measured by serum 8-OHdG [104]. Observations from in vitro studies indicate that hyperglycemia can cause DNA damage and mutation [105,106]. Hyperglycemia increases production of AGEs (advanced glycation end products) which promote DNA breaks and 8-OHdG accumulation [107,108]. High glucose levels may induce DNA damage in cells through AKT (Protein Kinase B) phosphorylation and tuberin phosphorylation [109,110]. The AKT pathway is involved in cell growth and DNA repair. AKT activation leads to inhibition of protein recruitment to DNA damage site and therefore disturbs homologous repair [111]. ROS-related AKT induction was associated with low expression of OGG1, a protein involved in repair of oxidative DNA lesion and resulted in the accumulation of DNA damage [109,112,113]. Also, *XPD* (Xeroderma Pigmentosum group D) gene involved in DNA repair by NER was down-regulated as a result of long-term exposure to high glucose concentration. Insulin was found to affect *XPD* gene expression via p70S6 kinase signaling pathway critical for cell-cycle progression and via RAS—a regulator of DNA damage checkpoint [114,115].

There is no doubt that obesity is associated with oxidative stress causing DNA damage. Repair of oxidized, saturated, and ring-fragmented bases via the BER pathway are known to be critical for maintaining genomic stability. On the other hand, an important role of DNA repair proteins in modulating mitochondrial energetics and whole-body energy balance was shown [116]. Products of such genes as OGG1, NTH1, NEIL1, and NEIL2 participate in the initiation of repair of oxidative DNA lesions. NEIL1 is an enzyme that initiates BER of ring-fragmented purines and some saturated pyrimidines [117,118]. The *neil1* knockout mice developed symptoms consistent with metabolic syndrome: severe obesity, fatty liver, dyslipidemia, and insulin resistance [119]. OGG1, a critical enzyme of the BER repair pathway, participates in the repair of the most common oxidative DNA lesion as 8-oxo-7,8-dihydroguanine (8-oxoG) and OGG1 expression is induced in response to a high-fat diet [120]. Mice lacking OGG1 (Ogg1−/−) developed features of metabolic syndrome, including increased adiposity, fatty liver, elevated triglycerides, and impaired glucose tolerance [116].

**Figure 3:**
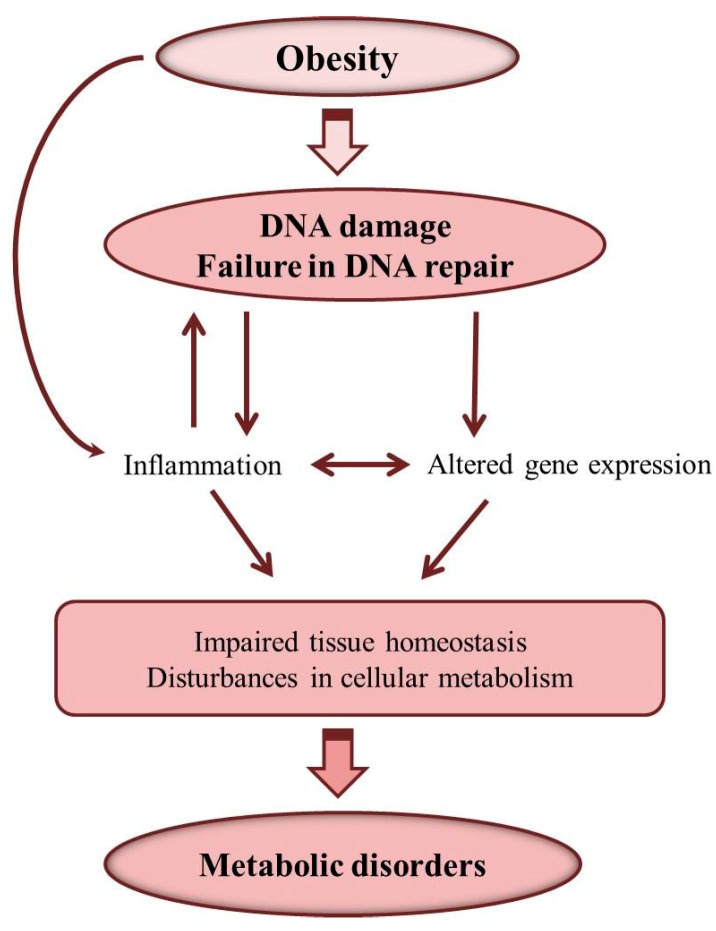
Obesity-induced DNA damage and development of metabolic disorders.

Therefore, obesity can induce DNA damage and disturbances in DNA repair resulting in cellular accumulation of DNA damage, which causes inflammation and alterations in gene expression and disturbances in cellular metabolism (Figure 3). As a consequence of these alterations, metabolic disorders can develop, and reduction of DNA damage may be important for the prevention and treatment of obesity-related metabolic diseases [5,121].

## 6. DNA Damage and Development of Obesity-Related Cancer

Obesity-induced DNA damage and dysregulation of the DNA repair pathways can lead to increased mutation rate and transformation of healthy tissues to cancer [25,122,123,124,125].

The International Agency for Research on Cancer has identified several cancers associated with overweight and obesity including postmenopausal breast cancer, endometrial cancer, renal cell carcinoma, esophageal adenocarcinoma, pancreatic, colorectal, and liver cancers [126,127,128,129,130]. About 55% of all cancers diagnosed in women and 24% of those diagnosed in men are associated with overweight and obesity [131]. Between 2005–2014 cancers associated with overweight and obesity, excluding colorectal cancer, increased 7%. Colorectal cancer incidence decreased by 23%, but this is due in large part to the screening. The pooled analysis of 42 prospective and 14 retrospective studies have shown that each increase in BMI by 5 kg/m^2^ was significantly associated with an 18% higher risk of colorectal cancer [132]. The meta-analysis of 82 studies on breast cancer (including 213,075 breast cancer survivors with 23,182 deaths) has shown that relative risks of mortality are 1.75 for pre-menopausal and 1.34 for postmenopausal breast cancer for obese women. Each 5 kg/m^2^ BMI increase before and after 1 year of cancer diagnosis increases risks by 18% and 29% for breast cancer mortality, respectively. In this case, obesity was associated with poorer breast cancer survival regardless of BMI ascertainment period [126]. Cancers associated with overweight and obesity, excluding colorectal cancer, increased among adults younger than 75 years. Moreover, there is an increase in the frequency of cancers associated with overweight and obesity (by 7%) in comparison to non-obesity cancers (13% drop).

**Figure 4:**
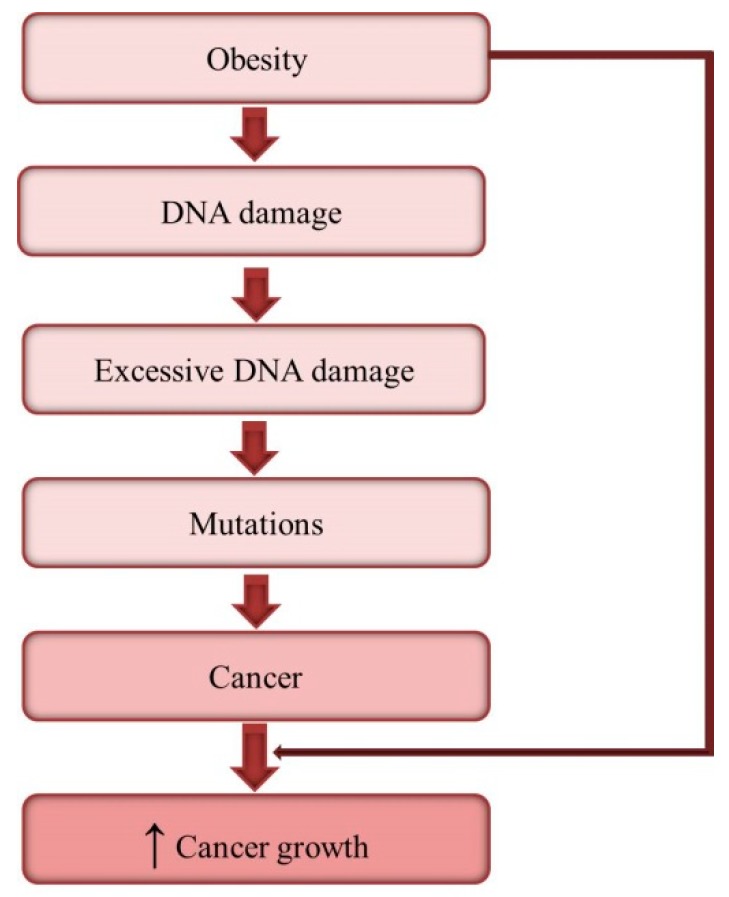
Obesity-induced DNA damage and cancer development.

Obesity-associated DNA damage cannot only enhance cancer risk but also promote cancer growth (Figure 4). DNA damage induced chronic inflammation, insulin resistance and alter gene expression can favor cancer cell proliferation and migration, resistance to apoptosis as well as tumor angiogenesis [133,134,135,136,137]. In addition, associated with obesity reduced secretion of adiponectin and increased secretion of leptin by adipose tissue can promote cancer development in obese. Adiponectin possesses anti-inflammatory and anti-angiogenic properties and can inhibit cancer growth [138]. Some tumor cells express adiponectin receptors, thus adiponectin by binding and activating these receptors can downstream signaling pathways in cancer cells and adiponectin deficiency excludes such action [139,140]. Leptin is a mitogenic, anti-apoptotic, pro-angiogenic, and proinflammatory factor [141]. Therefore, high leptin favors cancer growth and the relationship between circulating leptin concentrations and colorectal cancer risk has been demonstrated [142]. Obesity-associated abnormalities in the secretion of adipokines and cytokines lead to the activation of oncogenic intracellular molecular networks such as NF-κB, JAK2/STAT3 or PI3K/AKT pathways [143,144]. NF-κB signaling plays important role in modulating cancer cell response to DNA damage [145,146]. Hyperinsulinemia, commonly observed in obese, can reduce PI3K/AKT and affect p53 function. Gain-of-function p53 mutations enhance activation of AKT and, in turn, a modified response of cancer cells to insulin, leading to increased proliferation and migration [147].

The described above effect of DNA damage on the development of disturbances in glucose metabolism and hyperglycemia, commonly observed in obese, may promote tumor growth by providing cancer cells with energy and allowing them to maintain a rapid rate of cell division [148,149,150]. The high rate of glucose metabolism has been also reported to be associated with both activation of oncogenes and loss of tumor suppressors [150,151,152].

The accumulation of adipose tissue is a significant source of estrogens after the menopause. Enhance levels of estrogens can increase cell proliferation in the breast and uterus and increase the risk of cancer. ROS generation during estrogen metabolism may also promote oxidative DNA base damage [153]. Quinone and semiquinone metabolites of endogenous estrogens undergo redox cycling in breast epithelial cells, resulting in superoxide radical anion and H_2_O_2_ production [154,155]. In addition, 2,3-quinone and 3,4-quinone have the potential to initiate the cancer process by forming DNA adducts [156,157]. Estrogen signaling has been also recognized as a factor regulating DDR (DNA damage response) proteins such as ATM, ATR, p53, BRCA1, and BRCA2, as well as directly interacting with the DNA repair machinery [158,159]. Any disruption of DNA repair pathways may support cancer cell proliferation. Obesity-induced alterations in expression of proteins involve in DNA repair, such as PARP1, γH2AX, ATM, FANCD2, PTEN, BRCA1, and p53 were found to affect carcinogenesis and disease outcomes [160,161,162].

## 7. Conclusions

Obesity, caused mainly by chronic energy overload resulting from consumption of high-energy meals and reduced physical activity, and associated with oxidative stress and inflammation, has been recognized as a key factor inducing DNA damage and inhibiting DNA damage repair mechanisms, favoring accumulation of DNA damage, and leading to enhanced mutation rate and altering gene expression. Cellular response to DNA damage can result in irreversible cell-cycle arrest, activation of several proteins which can induce adipocyte differentiation and hypertrophy, inflammation, disturbances in cell metabolism, impair glucose metabolism, and promote the development of systemic insulin resistance. Accumulation of mutagenic DNA lesions is related to cancer development. In addition, obesity-associated metabolic disturbances and excessive DNA damage can promote cancer growth by favoring cancer cell proliferation and migration, and resistance to apoptosis. Estimation of the DNA damage and/or disturbances in DNA repair could be potentially useful in the early risk assessment and prevention of obesity-associated metabolic disorders as well as cancers since DNA damage in obesity appears to be reversible and both weight loss and improvement of dietary habits and diet composition can affect genome stability.

## Abbreviations

8-OHdG	8-OH deoxyguanosine
AGEs	advanced glycation end products
APE	apurinic endonuclease
ATM	ataxia telangiectasia–mutated protein
BER	base excision repair
BLM	bloom syndrome protein
BMI	body mass index
COX-2	cyclooxygenase 2
CSA	cockayne syndrome protein A
CSB	cockayne syndrome protein B
DAPI	4′,6-diamidino-2-phenylindole
DDR	DNA damage response
DFI	DNA fragmentation index
DNA-PK	DNA-dependent kinase
DR	direct repair
DSB	double-strand breaks
ELISA	enzyme-linked immunosorbent assay
ENDS	electronic nicotine delivery devices
EtBr	ethidium bromide
EXO1	human exonuclease 1
FANCF	fanconi anemia complementation group
Fapy Ade	4,6-diamino-5-formamidopyrimidine
Fapy Gua	2,6-diamino-4-hydroxy-5-formamidopyrimidine
FEN1	flap endonuclease 1
FFA	free fatty acids
FISH	fluorescence in situ hybridization
GC-MS	gas chromatography-mass spectrometry
GGR	global genome repair
H_2_O_2_	hydrogen peroxide
HPLC	high performance liquid chromatography
HR	homologous recombination
IL-6	interleukin 6
LIG	DNA ligase
MGMT	O6-methylguanine DNA methyltransferase
MN	micronucleus assay
MMR	mismatch repair
NADPH	nicotinamide adenine dinucleotide phosphate
NER	nucleotide excision repair
NF-κB	nuclear factor κB
NHEJ	non-homologous end-joining
NO	nitric oxide
NOS	nitric oxide synthase
NOX4	NADPH oxidase 4
OGG1	8-Oxoguanine glycosylase
PAH	polycyclic aromatic hydrocarbons
PARP	poly ADP-ribose polymerase 1
PCR	polymerase chain reaction
PCNA	proliferating cell nuclear antigen
PhIP	2-amino-1-methyl-6-phenylimidazo(4,5-b)pyridine
PTEN	phosphatase and tensin homolog
ROS	reactive oxygen species
RPA	replication protein A
SCGE	Single cell gel electrophoresis
SSB	single-strand breaks
ssDNA	single-strand DNA
SOD	superoxide dismutase
TCR	transcription-coupled repair
TFIIH	transcription factor complex IIH
TNFα	tumor necrosis factor
TUNEL	terminal deoxynucleotidyl transferase dUTP nick end labeling UV
UV	ultraviolet
WHO	World Health Organization
WRN	Werner syndrome protein
XP	xeroderma pigmentosium
XRCC	X-ray repair cross-complementing group
